# Hospital Expenditure and Efficiency

**Published:** 1905-03-18

**Authors:** 


### HOSPITAL ADMINISTRATION.

### CONSTRUCTION AND ECONOMICS.

### HOSPITAL EXPENDITURE AND EFFICIENCY.

#### CERTAIN FIGURES PUT TO THE TEST.

Scotch Hospitals.

Statements in regard to Scotch hospitals which have appeared in the Press as emanating from the London Hospital, with certain figures which have been quoted by Mr. Sydney Holland in recent speeches reported in the London papers, have caused some criticism in Scotland not entirely devoid of excitement. Col. Warburton, M.D., the Superintendent of the Royal Infirmary, Edinburgh, has issued a statement on the subject which will be found below. It will be seen that he disputes generally the accuracy of the conclusions drawn by Mr. Holland on several important points.

At the discussion on Dr. Mackintosh's paper before the Hospitals Association Mr. Sydney Holland quoted a number of figures from printed reports by the secretary, steward, and matron's assistant of the London Hospital of a visit made by them to certain Scotch and other hospitals. Dealing with Scotch hospitals Mr. Sydney Holland stated that Dr. Mackintosh could get home-grown Scotch meat at 3¾d per lb. At the London Hospital they had to pay 4¾d. or 5d. per lb. On this point Colonel Warburton states that home-fed Scotch beef and mutton cost the Edinburgh Royal Infirmary 6½d. per lb. as compared with 4¾d. or 5d. for foreign or colonial meat in the London Hospital. We have ascertained too from the Western Infirmary that, whereas mutton cost in the London Hospital 5d. and 6d. per pound, they pay 8d. for mutton at the Western Infirmary. Mr. Holland stated that in Glasgow they got their window cleaning done for £26, and that it cost £300 at the London. It should be mentioned that although £26 is the sum put down in the accounts of the Western Infirmary for window cleaning, the maids and porters do much of the window cleaning on the lower floors. Mr. Holland stated that, while the Glasgow Infirmary got their telephones free, at the London they paid £325 a year. Dr. Mackintosh however pointed out that although the telephone connection with the town system was free in Glasgow, if the Western paid for it it would cost them only £10 10s. a year instead of £325 which the London spends on telephones alone. Mr. Holland stated that the rates and taxes were £35 at the Western Infirmary, but on page 15 of the last annual report it is shown that the amounts paid for rates and taxes for the year was in reality £237 12s. 9d. Mr. Holland further stated that in Scotland they gave no pensions, whilst at the London they pay £2,200 a year in pensions. At the Western Infirmary no pensions are yet paid because it is a comparatively new hospital, but large sums are paid in pensions at the older Scotch hospitals like the Royal Infirmary, Glasgow, and the Royal Infirmary, Edinburgh. Mr. Holland stated that Dr. Mackintosh did no massage, whilst at the London they had 13,000 cases in the out-patient department alone. As a matter of fact on page 17 of the Western Infirmary report it is shown that £47 19s. 6d. was spent for massage during the year, a sum which is quite apart from the massage which is undertaken by the regular staff of the hospital. Mr. Holland appeared to be under the impression too that an out-patient department at the Western Infirmary was something quite new, which is not the case; nor is it the case that no medicine is given to out-patients, for, as a matter of fact 39,974 prescriptions were dispensed last year to out-patients at the Western Infirmary.

Guy's Hospital.

Mr. Sydney Holland stated that Guy's Hospital "in provisions, in alcohol, in surgery, and in domestic service was behind other London hospitals," and that it "was not a pattern to the rest for economy." Mr. Holland should have been aware that taking the figures for the year 1903 Guy's Hospital in regard to its expenditure on surgery and domestic expenses, and on salaries and wages, is much below the average cost of the 16 principal voluntary hospitals in London, and that in regard to provisions the average cost per bed at Guy's is only about 3s. higher than the average cost under this head of the 16 hospitals in question. Anyone who takes the trouble to examine the figures in "Burdett's Hospitals and Charities" for 1905 will find that the results for the year 1903 work out as follows:—

| Items. | Cost per Bed at Guy's. | Average Cost per Bed at 12 London Hospitals with Medical Schools. |
|---|---|---|
| | £ s. d. | £ s. d. |
| Provisions... ... | 28 15 0 | 27 2 6 |
| Alcohol ... ... | 0 15 0 | 1 5 5 |
| Surgery ... ... | 13 15 0 | 14 17 6 |
| Domestic expenses | 20 15 0 | 25 11 8 |
| Salaries and wages | 27 5 0 | 32 14 2 |

It will thus be seen that several of the statements made in the discussion at the Hospitals Association by Mr. Sydney Holland in regard to Guy's Hospital are not maintainable when the actual figures under the various headings quoted are examined in detail. For with the exception of provisions where the cost per occupied bed at Guy's Hospital is £1 12s. 6d. above the average of the 12 London Hospitals with medical schools, and in the case of salaries and wages at King's College and the Royal Free Hospitals where the cost of this item was £6 5s. and £2 5s. per bed respectively less than at Guy's; the expenditure at Guy's Hospital is considerably less than the average. The expenditure on salaries and wages at the London Hospital was £41 10s. per bed occupied compared with £27 5s. at Guy's Hospital, and it is not reasonable to compare an item like this in regard to relatively small hospitals like King's College, which had only 184 beds occupied, and the Royal Free Hospital with 145 occupied beds, with hospitals like Guy's where the occupied beds amounted to 499 and the London to 676 in the year 1903.

The moral of the errors here corrected is of course simple. It is essential that everybody who makes comparisons of this kind should be careful to ascertain the facts and to reduce any figures which may be used to a uniform basis, so that the deductions drawn may rest upon ascertained facts, which are incontrovertible, and may be wholly divorced from prejudice, or a not unnatural desire perhaps to score a point when speaking at a public meeting.

"A Mistake."

The following statement was included in the report on the hospitals visited, which was signed by the Secretary, the Steward and the Matron's Assistant of the London Hospital :—

"And it seems strange that Sir Henry Burdett should have quoted the cost per bed of this hospital (the Royal Infirmary, Glasgow) as an example to London hospitals. He cannot have known anything about the condition of the hospital."

Sir Henry Burdett having asked for an explanation, has received a letter admitting that the above statements are without foundation, together with an assurance that every person to whom a copy of the report has been sent has been informed of this mistake, and that the reference to Sir Henry Burdett had been inserted in error.

